# Association of alcohol intake over the lifetime with colorectal adenoma and colorectal cancer risk in the Prostate, Lung, Colorectal, and Ovarian Cancer Screening Trial

**DOI:** 10.1002/cncr.70201

**Published:** 2026-01-26

**Authors:** Caitlin P. O’Connell, Sonja I. Berndt, Kenechukwu Chudy‐Onwugaje, Andrew Kunzmann, Wen‐Yi Huang, Kathryn Hughes Barry, Erikka Loftfield

**Affiliations:** ^1^ Division of Cancer Epidemiology and Genetics Metabolic Epidemiology Branch National Cancer Institute National Institutes of Health Rockville Maryland USA; ^2^ Division of Cancer Epidemiology and Genetics Occupational and Environmental Epidemiology Branch National Cancer Institute National Institutes of Health Rockville Maryland USA; ^3^ Center for Global Health Perelman School of Medicine University of Pennsylvania Philadelphia Pennsylvania USA; ^4^ Wellcome–Wolfson Institute for Experimental Medicine Queen’s University Belfast Belfast UK; ^5^ Department of Epidemiology and Public Health University of Maryland School of Medicine Baltimore Maryland USA

**Keywords:** adenomatous polyps, alcohol abstinence, alcohol drinking, alcoholic beverages, colon cancer, colorectal cancer, colorectal neoplasms, drinking behavior, rectal cancer

## Abstract

**Background:**

Alcohol drinking is associated with higher colorectal cancer (CRC) risk, but research on lifetime alcohol drinking is limited. The objective of the current study was to estimate the association of lifetime alcohol drinking with incident colorectal adenoma and cancer.

**Methods:**

US adults enrolled in the Prostate, Lung, Colorectal, and Ovarian Cancer Screening Trial reported alcohol intake during four age periods. Average lifetime alcohol intake was calculated as average drinks per week from age 18 years until study baseline. Alcohol intake patterns were defined by past and current drinking frequency. Among 12,327 participants with a negative baseline screen, 812 had an adenoma on the second screen. Logistic regression was used to estimate odds ratios (ORs) and 95% confidence intervals (CIs) for incident adenoma. During 20 years of follow‐up, 1679 incident CRC cases occurred among 88,092 participants. Cox proportional hazards regression was used to estimate hazard ratios (HRs) and 95% CIs for CRC.

**Results:**

Current drinkers with an average lifetime alcohol intake of 14 or more drinks per week, compared with one drink or less per week, had a higher risk of CRC (HR, 1.25; 95% CI, 1.01–1.53), especially rectal cancer (HR, 1.95; 95% CI, 1.17–3.28). Consistent heavy drinking versus light drinking was positively associated with CRC risk (HR, 1.91; 95% CI, 1.17–3.12). Compared with current drinkers averaging less than one drink per week, former drinkers had lower odds of nonadvanced adenoma (OR, 0.58; 95% CI, 0.39–0.84). Current drinkers averaging from seven to less than 14 drinks compared with less than one drink per week had a lower risk of CRC (HR, 0.79; 95% CI, 0.64–0.97), especially distal colon cancer (HR, 0.64; 95% CI, 0.42–1.00).

**Conclusions:**

Consistent heavy alcohol intake and higher average lifetime alcohol drinking may increase CRC risk, whereas cessation may lower adenoma risk. Associations may differ by tumor site.

## Abstract 2

In the Prostate, Lung, Colorectal, and Ovarian Cancer Screening Trial cohort, participants who drank 14 or more alcoholic drinks per week, compared with those who drank less than one alcoholic drink per week, on average, throughout adulthood, and participants who reported heavy drinking across all age ranges had higher risks of colorectal cancer, especially rectal cancer. Former drinkers had lower odds of nonadvanced adenomas.

## INTRODUCTION

Colorectal cancer (CRC) is the third most common cancer type in the United States.^1^ Overall, CRC incidence has declined in the United States; however, among adults younger than 55 years, CRC incidence has increased since the mid‐1990s.^1^, ^2^ The US Surgeon General’s Advisory recently reported that alcohol drinking is a leading preventable cause of cancer and highlighted the association with CRC risk.^3^ The International Agency for Research on Cancer has determined that alcoholic beverages are *carcinogenic to humans* and causally related to CRC.^4^ Much of the epidemiologic evidence supporting this determination comes from cohort studies that measured recent (e.g., in the previous 12 months) alcohol drinking at study baseline.^5^, ^6^, ^7^ Prospective studies that have explored lifetime alcohol drinking or changes in drinking and CRC risk^8^, ^9^, ^10^, ^11^, ^12^, ^13^ have generally reported a positive association between higher lifetime consumption and higher CRC risk. However, findings have been less consistent by anatomic location of the tumor. Although the literature is mixed on the association of current alcohol intake and colorectal adenoma,^14^, ^15^, ^16^, ^17^ research on lifetime alcohol drinking with incident adenoma, a precursor to CRC, is sparse. One study on prevalent adenoma found no association,^18^ whereas a prospective study on incident adenoma found a positive association.^19^

Herein, we leveraged data on alcohol intake reported for multiple age groups from the prospective Prostate, Lung, Colorectal, and Ovarian (PLCO) Cancer Screening Trial to evaluate the effect of lifetime alcohol intake on both incident adenoma and CRC risk within the same prospective study. The PLCO trial has the following five ClinicalTrials.gov registration numbers: NCT00002540 (prostate), NCT01696968 (lung), NCT01696981 (colorectal), NCT01696994 (ovarian), and NCT00339495 (etiology and early marker studies).

## MATERIALS AND METHODS

### Study population

Participants in this study were adults enrolled in the PLCO Cancer Screening Trial, a randomized, controlled trial of men and women designed to assess whether cancer screening reduced mortality from four cancers. The design of the PLCO Cancer Screening Trial has been previously described in detail.^20^ Briefly, in total, 154,887 participants, aged 55–74 years with no history of prostate, lung, colorectal, or ovarian cancer were enrolled between 1993 and 2001 across 10 screening centers in the United States. Participants who were randomized to the screening arm of the trial (*n* = 77,443) underwent flexible sigmoidoscopy at baseline and three years (T3) or five years (T5) later; those who were randomized to the control arm received standard of care.^21^ Participants completed a risk factor questionnaire at baseline, which captured demographic, medical, and lifestyle information, and a dietary history questionnaire (DHQ) at baseline (control arm) or approximately 3 years after baseline (screening arm). All participants gave informed consent at study enrollment. The study was approved by the Institutional Review Board at the National Cancer Institute and at each of the 10 study centers.

### Incident colorectal adenoma

For the incident adenoma analysis, participants were limited to those randomized to the screening arm who had a negative flexible sigmoidoscopy screen at baseline and an adequate second sigmoidoscopy examination at T3/T5 (*n* = 22,521). We further excluded participants who did not complete the baseline questionnaire (*n* = 21); who self‐reported having a prior colorectal polyp (*n* = 1196) or colorectal disease (i.e., ulcerative colitis, Crohn disease, Gardner syndrome, or familial polyposis; *n* = 365); who had an invalid DHQ, which was defined as missing a completion date, missing responses for eight or more 8 items, measuring calorie intake in the first or 99th percentile of intake, or being completed after date of death (*n* = 3372); who had filled out the DHQ after the T3/T5 screen (*n* = 4622); and current drinkers who had missing lifetime alcohol data (*n* = 618).

Cases (*n* = 812) were defined as participants who had a negative screen at enrollment and a positive screen that was determined to be adenoma in the distal colon or rectum at T3/T5. Participants with a positive sigmoidoscopy screen were referred to their primary physician for follow‐up colonoscopy, and medical records were abstracted for diagnoses. Controls (*n* = 11,515) were defined as those who had a negative screen both at baseline and at T3/T5. Cases were further classified by adenoma severity (advanced, nonadvanced, and unknown) and anatomic location (rectum, distal colon, both, and unknown). Advanced adenoma (*n* = 197) was defined as adenoma with villous or tubular‐villous characteristics, large size (≥1 cm), or severe dysplasia.^22^, ^23^ Our final incident adenoma analytic cohort included 12,327 participants.

### Incident CRC

For the CRC analysis, our population included participants in both arms of the study who had no personal history of prostate, lung, colorectal, or ovarian cancer (*N* = 154,887). We excluded participants who did not complete a baseline questionnaire (*n* = 4918), who had an invalid DHQ (*n* = 38,462), who had a history of CRC before the DHQ (*n* = 474), who self‐reported a colorectal disease (*n* = 2471), who had no follow‐up time (*n* = 51), who did not consent to further follow‐up at trial close‐out (*N* = 14,193), and those missing lifetime alcohol data (*N* = 6226). Our final CRC analytic cohort included 88,092 participants.

CRC diagnoses were ascertained from state cancer registries or self‐report and confirmed through abstraction of medical records through 2017.^24^ CRC was defined using the following International Classification of Diseases for Oncology, second edition, site codes: C180, C182–C189, C199, C209, C212, and C218. Cases were further classified by anatomic location (rectum, distal colon, proximal colon, and unclear). Follow‐up time was calculated from date of DHQ completion to the date of CRC diagnosis, death, withdrawal from the study, or the end of follow‐up for cancer incidence (December 31, 2017).

### Exposure variable

Lifetime alcohol use was assessed using the DHQ. Participants were asked about frequency of consumption of beer, wine, and liquor during four predefined age ranges (18–24, 25–39, and 40–54 years and 55 years and older ) using 10 predefined frequency categories from never to six or more drinks per day. Participants were also asked about their beer, wine, and liquor consumption in the past year on the DHQ. Lifetime alcohol intake was calculated as a weighted average: the sum of the number of alcoholic drinks per day multiplied by the number of years in that age range and then divided by the total number of years from 18 to age at the date of DHQ completion (see Supporting Methods). We further categorized participants into never drinkers, former drinkers, and current drinkers (i.e., reported drinking on the DHQ) with the following categories of average lifetime drinking: less than one, from one to less than seven, from seven to less than 14, and 14 or more drinks per week. In addition, we used past alcohol intake to define drinking patterns across adulthood. We categorized participants by drinking status (never, former, current) and frequency. We used the sex‐specific US Dietary Guidelines of limiting alcoholic drinks to one drink per day or less for women and two drinks per day or less for men^25^ to classify past and current drinking as light (less than seven drinks per week for women and less than 14 drinks per week for men), moderate (from seven or more to 14 or less drinks per week for women and from 14 or more to 21 or less drinks per week for men), and heavy (greater than 14 drinks per week for women and greater than 21 drinks per week for men; see Supporting Methods).

### Statistical analysis

We tabulated demographic and lifestyle factors by lifetime alcohol drinking. For analyses of incident adenoma, we used logistic regression models to estimate odds ratios (ORs) and 95% confidence intervals (CIs) for lifetime alcohol drinking and, in separate models, the alcohol drinking patterns. We used multinominal logistic regression to estimate associations by adenoma advanced status and anatomic location. We used Cox proportional hazards regression models to estimate hazard ratios (HRs) and 95% CIs for the average lifetime alcohol drinking and drinking patterns with CRC incidence. In our primary analyses, we used current drinkers with the lowest lifetime alcohol use as our reference group to minimize any potential bias arising from health and lifestyle differences between drinkers and lifetime abstainers^26^, ^27^, ^28^, ^29^ and because of the smaller numbers of never drinkers, which could lead to unstable effect estimates. However, we performed secondary analyses with never drinkers as the reference group. Person‐years of follow‐up were used as the underlying time metric. Results with age as the underlying time metric were similar. We used Schoenfeld residuals to assess the proportional hazard assumption. To calculate HR estimates and *p* values for heterogeneity for each level of lifetime alcohol drinking and CRC risk by anatomic location, we performed a competing‐risks survival analysis using a cause‐specific Cox proportional hazards regression model^30^, ^31^ for the three CRC subsites combined. CRC cases with unknown or multiple locations (*n* = 38) were excluded from the anatomic location analysis.

We adjusted all models for potential confounding factors, which were identified a priori based on the literature and the unique PLCO study design, including age, sex, screening year (adenoma only), trial arm (CRC only), body mass index (BMI), race/ethnicity, education, smoking status, family CRC history, diabetes history, nonsteroidal anti‐inflammatory drug use, hormone‐replacement therapy (females only), and intake of energy, dietary calcium, fiber, folate, and red meat. We used the nutrient density approach^32^ to energy‐adjust dietary variables and scaled them to reduce multicollinearity. We used indicator variables to account for missing categorical data. No variable had more than 3.1% missing data. To evaluate whether each covariate appreciably altered effect estimates, we calculated the percentage change in the beta coefficients for lifetime alcohol drinking with adenoma and CRC when adding each covariate to the age‐adjusted and sex‐adjusted model; and the following covariates altered effect estimates by ≥10%: BMI, race, smoking, education, diabetes, and calcium, fiber, and folate intake.

We used restricted cubic spline models to assess potential nonlinear associations between lifetime alcohol drinking and CRC risk among current drinkers. We evaluated potential effect modification by sex and trial arm (CRC only) using the likelihood ratio test to compare models with and without an interaction term between sex or trial arm and lifetime alcohol intake. We ran two CRC sensitivity analyses: (1) excluding those with <2 years of follow‐up and (2) removing the adjustment for BMI that may lie on the causal pathway. A *p* value < .05 was considered statistically significant. All statistical analyses were conducted using R version 4.3.3 (R Foundation for Statistical Computing).

## RESULTS

### Population characteristics

In the PLCO Cancer Screening Trial, most participants were current drinkers (73.4%; Table 1). Baseline age was slightly older among never drinkers compared with current drinkers. Compared with current drinkers in the lowest (less than one drink per week), participants in the highest (14 or more drinks per week) average lifetime drinking category were more likely to be male (89.8% vs. 24.2%), non‐Hispanic White (90.7% vs. 90.2%), current smokers (18.3% vs. 6.4%), and overweight (48.1% vs. 38.2%); they were also less likely to be college educated (34.7% vs. 37.6%). Heavier drinkers tended to eat less calcium, fiber, and folate compared with lighter drinkers. Descriptive statistics were similar in the incident adenoma subcohort (Table S1).

**TABLE 1:** Baseline characteristics by lifetime alcohol intake in the Prostate, Lung, Colorectal, and Ovarian Cancer Screening Trial cohort (*N* = 88,092).

Characteristic	Lifetime average drinking category, No. (%)					
			Current drinker			
	Never drinker	Former drinker	<1 drink/week	1 to <7 drinks/week	From 7 to <14 drinks/week	≥14 drinks/week
No. of participants	10,205	13,207	15,621	33,267	9069	6723
Lifetime alcohol intake: Median [IQR], drinks/week	0.0 [0.0–0.0]	0.9 [0.3–6.2]	0.53 [0.3–0.7]	2.99 [1.83–4.6]	9.43 [8.0–11.4]	20.19 [16.4–27.7]
Age at DHQ: Median [IQR], years	67.0 [62.0–71.0]	65.0 [61.0–70.0]	65.0 [60.0–70.0]	65.0 [60.0–70.0]	65.0 [61.0–70.0]	65.0 [60.0–69.0]
Sex						
Male	3191 (31.3)	6778 (51.3)	3784 (24.2)	17,138 (51.5)	6839 (75.4)	6038 (89.8)
Female	7014 (68.7)	6429 (48.7)	11,837 (75.8)	16,129 (48.5)	2230 (24.6)	685 (10.2)
Race/ethnicity						
Asian	805 (7.9)	581 (4.4)	717 (4.6)	719 (2.2)	212 (2.3)	234 (3.5)
Hispanic	95 (0.9)	198 (1.5)	209 (1.3)	485 (1.5)	130 (1.4)	98 (1.5)
Non‐Hispanic Black	543 (5.3)	760 (5.8)	510 (3.3)	801 (2.4)	198 (2.2)	211 (3.1)
Non‐Hispanic White	8669 (84.9)	11,537 (87.4)	14,091 (90.2)	31,095 (93.5)	8458 (93.3)	6101 (90.7)
Other/unknown	93 (0.9)	131 (1.0)	94 (0.6)	167 (0.5)	71 (0.8)	79 (1.2)
College graduate or higher	3497 (34.3)	3920 (29.7)	5874 (37.6)	14,221 (42.7)	4024 (44.4)	2331 (34.7)
BMI category, kg/m^2^						
<18.5	101 (1.0)	100 (0.8)	115 (0.7)	193 (0.6)	51 (0.6)	25 (0.4)
18.5 to <25.0	3548 (34.8)	3779 (28.6)	5664 (36.3)	11,933 (35.9)	2843 (31.3)	1780 (26.5)
25.0–30.0	3928 (38.5)	5461 (41.3)	5972 (38.2)	14,210 (42.7)	4271 (47.1)	3232 (48.1)
≥30	2512 (24.6)	3710 (28.1)	3675 (23.5)	6533 (19.6)	1815 (20.0)	1591 (23.7)
Smoking status						
Never smoker	8811 (86.3)	5206 (39.4)	9908 (63.4)	13,747 (41.3)	2474 (27.3)	1306 (19.4)
Former smoker	1119 (11.0)	6455 (48.9)	4711 (30.2)	16,446 (49.4)	5502 (60.7)	4185 (62.2)
Current smoker	274 (2.7)	1544 (11.7)	1002 (6.4)	3070 (9.2)	1093 (12.1)	1230 (18.3)
Family history of colon cancer	1040 (10.2)	1362 (10.3)	1641 (10.5)	3301 (9.9)	862 (9.5)	589 (8.8)
Regular NSAID use	4477 (43.9)	6620 (50.1)	7355 (47.1)	16,893 (50.8)	4900 (54.0)	3457 (51.4)
History of diabetes	988 (9.7)	1687 (12.8)	823 (5.3)	1500 (4.5)	458 (5.1)	482 (7.2)
HRT use	4496 (44.1)	4149 (31.4)	8046 (51.5)	11,438 (34.4)	1592 (17.6)	467 (6.9)
Total energy intake: Median [IQR], kcal/day	1470 [1120–1920]	1590 [1200–2100]	1430 [1100–1840]	1610 [1250–2060]	1900 [1490–2380]	2190 [1690–2840]
Dietary calcium intake: Median [IQR], mg/1000 kcal/day	430 [337–567]	412 [327–537]	423 [337–547]	404 [326–516]	368 [298–463]	334 [266–425]
Red meat intake: Median [IQR], g/1000 kcal/day	25.4 [15.2–38.4]	30.3 [18.4–45.2]	27.9 [17.5–40.4]	31.5 [20.4–45.1]	34.7 [23.1–49.1]	35.5 [23.9–50.5]
Dietary fiber intake: Median [IQR], g/1000 kcal/day	11.1 [9.01–13.8]	10.5 [8.35–13.1]	11.0 [8.92–13.4]	10.1 [8.34–12.3]	9.20 [7.57–11.2]	8.49 [6.96–10.4]
Dietary folate intake: Median [IQR], μg/1000 kcal/day	228 [192–273]	219 [182–265]	228 [192–271]	215 [182–255]	199 [167–237]	183 [153–221]
Trial arm						
Screening	5351 (52.4)	6916 (52.4)	8040 (51.5)	17547 (52.7)	4746 (52.3)	3522 (52.4)
Control	4854 (47.6)	6291 (47.6)	7581 (48.5)	15720 (47.3)	4323 (47.7)	3201 (47.6)

*Note:* Missing values are not shown. Percentages are column percents and do not always add up because of rounding and missing values.

Abbreviations: BMI, body mass index; DHQ, Dietary History Questionnaire; HRT, hormone‐replacement therapy; IQR, interquartile range; NSAID, nonsteroidal anti‐inflammatory drug.

### Incident adenoma analysis

Of the 12,327 participants who had a negative baseline CRC screen, 812 had an incident colorectal adenoma. Overall, we observed no significant association for current drinkers with higher, compared with the lowest, average lifetime intake, for higher adenoma risk (Table 2). However, a suggestive inverse association was observed for former drinkers compared with current drinkers who had an average lifetime alcohol intake of less than one drink per week, (OR, 0.78; 95% CI, 0.59–1.02), and the association for nonadvanced adenoma (OR, 0.58; 95% CI, 0.39–0.84) reached statistical significance. We found no evidence of effect modification by sex (likelihood ratio test, *p* = .25; see Table S2). Alcohol intake pattern results were also similar (Table 3), and use of never drinkers as the reference group (see Table S3) did not appreciably alter findings. In both analyses, OR estimates were below 1.0 for drinking cessation and above 1.0, with increasing magnitude, for categories of current drinking, although these findings were not statistically significant.

**TABLE 2:** Odds ratios and 95% confidence intervals for average lifetime alcohol intake and incident adenoma by subtype and anatomic location.

	Overall				Adenoma severity^a^							Anatomic location^b^						
Alcohol drinking status	Any adenoma				Advanced adenoma			Nonadvanced adenoma				Rectal adenoma			Distal colon adenoma			
	Non cases, no.	Cases, no.	OR^c^	95% CI	Cases, no.	OR^c^	95% CI	Cases, no.	OR^c^	95% CI	*p*_het_ ^d^	Cases, no.	OR^c^	95% CI	Cases, no.	OR^c^	95% CI	*p*_he_ ^d^
Never	1441	71	0.82	0.60–1.10	15	0.85	0.44–1.63	47	0.93	0.64–1.35	.81	12	0.68	0.34–1.37	55	0.86	0.61–1.21	.56
Former	1668	106	0.78	0.59–1.02	29	1.08	0.62–1.90	48	0.58	0.39–0.84	.06	23	0.92	0.51–1.64	77	0.77	0.56–1.06	.61
Current: Lifetime average drinks/week																		
>0 to <1	2139	133	1.00	Ref	25	1.00	Ref	79	1.00	Ref	—	26	1.00	Ref	97	1.00	Ref	—
1 to <7	4424	312	0.92	0.74–1.14	75	1.13	0.70–1.81	164	0.79	0.59–1.05	.20	76	1.24	0.77–1.97	214	0.86	0.66–1.11	.17
7 to <14	1093	10	1.00	0.75–1.34	28	1.32	0.74–2.37	49	0.78	0.52–1.15	.13	21	1.21	0.65–2.25	77	1.02	0.73–1.42	.64
≥14	750	88	1.10	0.80–1.51	25	1.37	0.73–2.55	45	0.94	0.62–1.42	.32	21	1.57	0.82–3.01	57	0.96	0.66–1.40	.19

Abbreviations: CI, confidence interval; OR, odds ratio; *p*_het_, *p* for heterogeneity; Ref, reference group.

^a^ Results by adenoma severity were estimated using a multinomial logistic regression model with outcome categories of no adenoma (reference group), advanced adenoma, nonadvanced adenoma, and unknown severity. Estimates for unknown severity are not shown.

^b^ Results by anatomic location were estimated using a multinomial logistic regression model with outcome categories of no adenoma (reference group), rectal adenoma, distal colon adenoma, both rectal and distal adenoma, and adenoma in unknown location. Estimates for both and for unknown location are not shown.

^c^ Adjusted for sex (male or female), age (years), screening year (3 or 5 years after baseline), race (Asian, Hispanic, Non‐Hispanic Black, Non‐Hispanic White, or other/unknown), college graduate (yes/no), body mass index category (<18.5, 18.5 to <25.0, 25.0 to <30.0, or ≥30 kg/m^2^), smoking status (never smoker; cigar/pipe smoker only; former smoker, stopped >20 years ago; former smoker, stopped <20 years ago; current smoker, 0–44 pack‐years; current smoker, ≥44 pack‐years), regular nonsteroidal anti‐inflammatory drug use (yes/no), history of diabetes (yes/no), hormone‐replacement therapy use (females only; yes/no), family history of colon cancer (yes/no), total daily energy (kcal/day), dietary calcium intake (mg/1000 kcal/day), red meat intake (g/1000 kcal/day), dietary fiber intake (g/1000 kcal/day), and dietary folate intake (μg/1000 kcal/day). Overall global *p* value for average lifetime alcohol intake variable, *p* = .21; severity, global *p* = .15; anatomic location, global *p* = .52.

^d^ The *p*_het_ for advanced versus nonadvanced or rectal versus distal colon from multinomial logistic regression model.

**TABLE 3:** Odds ratios and 95% confidence intervals for lifetime alcohol intake patterns and incident adenoma.

Drinking status	Drinking frequency	Past range, drinks/week^a^	Current range, drinks/week^b^	Noncases, no.	Cases, no.	OR (95% CI)^c^
Never	Never	0	0	1441	71	0.86 (0.66–1.14)
Former	Former	Any value	0	1669	106	0.83 (0.66–1.04)
Current	Always light (below dietary guidelines^d^)	Women, >0 to <7 Men, >0 to <14	Women, >0 to <7 Men, >0 to <14	5896	407	1.00 (Ref)
	Occasionally moderate	At least at one point in time: Women, ≥7 to ≤14 Men, ≥14 to ≤21		1214	96	1.06 (0.84–1.35)
	Occasionally heavy	At least at one point in time: Women, >14 Men, >21		1236	125	1.13 (0.91–1.42)
	Always moderate or heavy	Women, ≥7 Men, ≥14	Women, ≥7 Men, ≥14	59	7	1.26 (0.56–2.85)

Abbreviations: CI, confidence interval; OR, odds ratio.

^a^ Participants were asked about the frequency of consumption of alcohol during four predefined age ranges (18–24, 25–39, 40–54, and ≥55 years).

^b^ Participants were asked about their alcohol consumption in the year before Dietary History Questionnaire completion.

^c^ Adjusted for sex (male or female), age (years), screening year (3 or 5 years after baseline), race (Asian, Hispanic, Non‐Hispanic Black, Non‐Hispanic White, or other/unknown), college graduate (yes/no), body mass index category (<18.5, 18.5 to <25.0, 25.0 to <30.0, ≥30.0 kg/m^2^), smoking status (never smoker; cigar/pipe smoker only; former smoker, stopped >20 years ago; former smoker, stopped <20 years ago; current smoker, 0–44 pack‐years; current smoker, ≥44 pack‐years), regular nonsteroidal anti‐inflammatory drug use (yes/no), history of diabetes (yes/no), family history of colon cancer (yes/no), total daily energy (kcal/day), dietary calcium intake (mg/1000 kcal/day), red meat intake (g/1000 kcal/day), dietary fiber intake (g/1000 kcal/day), and dietary folate intake (μg/1000 kcal/day). Global *p* value for lifetime alcohol intake pattern variable: *p* = .25.

^d^ US dietary guidelines for alcohol intake are one drink per day or less for women and two drinks per day or less for men.

### Colorectal cancer analysis

Among 88,092 participants with a median follow‐up time of 14.5 years, 1679 incident CRC cases occurred (247 rectal, 390 distal colon, 1004 proximal colon, and 38 unknown/multiple anatomic locations). Overall, we observed a positive association between current drinkers who had the highest average lifetime alcohol drinking compared with current drinkers who had the lowest average lifetime intake and CRC risk in the multivariable‐adjusted models (14 or more drinks vs. less than one drink per week: HR, 1.25; 95% CI, 1.01–1.53; *p* = .003; Table 4). Specifically, estimates for rectal cancer were positive, revealing a higher risk of rectal cancer among current drinkers with the highest average lifetime intake (14 or drinks more vs. less than one drink per week: HR, 1.95; 95% CI, 1.17–3.28; *p* for heterogeneity = .05). When stratified by trial arm, the positive HR estimate for 14 or more alcoholic drinks per week was strengthened in the control arm (HR, 1.39; 95% CI, 1.06–1.82; likelihood ratio test, *p* = .33; see Table S4). We also observed an inverse association between current drinkers with average lifetime alcohol drinking of from seven to less than 14 drinks per week compared with less than one drink per week and CRC risk (HR, 0.79; 95% CI, 0.64–0.97). The inverse association was strongest for distal colon cancer (from seven to less than 14 drinks vs. less than one drink per week: HR, 0.64; 95% CI, 0.42–1.00; *p* for heterogeneity = .30) and among those in the screening arm (from seven to less than 14 drinks vs. less than one drink per week: HR, 0.71; 95% CI, 0.51–0.98). Our restricted cubic spline analysis showed limited evidence for a nonlinear association (*p* for nonlinearity = .07) of lifetime alcohol drinking among current drinkers.

**TABLE 4:** Hazard ratios and 95% confidence intervals for average lifetime alcohol intake and incident colorectal cancer overall and by anatomic site.

Alcohol drinking status	Overall				Rectal				Distal colon				Proximal colon^d^			
	Person‐years	Cases, no.	HR^a^	95% CI	Cases, no.	HR^a^	95% CI	*p*_het_ ^b^	Cases, no.	HR^a^	95% CI	*p*_het_ ^c^	Cases, no.	HR^a^	95% CI	*p*_het_
Never	147,929	174	0.92	0.76–1.11	20	0.88	0.50–1.54	.88	30	0.71	0.46–1.11	.23	119	0.99	0.78–1.26	.25
Former	181,050	261	1.00	0.84–1.19	33	0.99	0.60–1.62	.96	72	1.08	0.76–1.53	.59	152	0.97	0.77–1.21	.64
Current: Lifetime average drinks/week																
>0 to <1	234,828	286	1.00	Ref	34	1.00	Ref	—	65	1.00	Ref	—	182	1.00	Ref	—
1 to <7	491,379	631	0.97	0.84–1.13	94	1.15	0.77–1.73	.32	140	0.86	0.63–1.16	.39	379	0.96	0.80–1.16	.98
7 to <14	130,091	149	0.79	0.64–0.97	27	1.08	0.63–1.84	.21	34	0.64	0.42–1.00	.30	86	0.79	0.60–1.03	.99
≥14	91,288	178	1.25	1.01–1.53	39	1.95	1.17–3.28	.05	49	1.16	0.77–1.75	.75	86	1.08	0.82–1.44	.21

Abbreviations: CI, confidence interval; OR, odds ratio; *p*_het_, *p* for heterogeneity; Ref, reference group.

^a^ Adjusted for sex (male or female), age (years), race (Asian, Hispanic, Non‐Hispanic Black, Non‐Hispanic White, or Other/unknown), college graduate (yes/no), BMI category (<18.5, 18.5 to <25, 25 to <30, ≥30 kg/m^2^), smoking status (never smoker; cigar/pipe smoker only; former smoker, stopped over 20 years ago; former smoker, stopped under 20 years ago; current smoker, 0−44 pack‐years; current smoker, ≥44 pack‐years), regular NSAID use (yes/no), history of diabetes (yes/no), family history of colon cancer (yes/no), total daily energy (kcal/day), dietary calcium intake (mg/1000 kcal/day), red meat intake (g/1000 kcal/day), dietary fiber intake (g/1000 kcal/day), dietary folate intake (ug/1000 kcal/day), and trial arm (intervention or control). Global *p* values for average lifetime alcohol intake: overall, *p* = .003; rectal, *p* = .07; distal colon, *p* = .04; proximal colon, *p* = .39.

^b^ The *p*_het_ for rectal versus distal and proximal from cause‐specific Cox proportional hazards regression model for the three colorectal cancer subsites combined.

^c^ The *p*_het_ for distal versus rectal and proximal from cause‐specific Cox proportional hazards regression model for the three colorectal cancer subsites combined.

^d^ The *p*_het_ for proximal versus distal and rectal from cause‐specific Cox proportional hazards regression model for the three colorectal cancer subsites combined.

We did not observe evidence of effect modification by sex (likelihood ratio test, *p* = .10; see Table S5). HR estimates overall and by trial arm were similar when we excluded the first 2 years of follow‐up (see Table S6) and when we did not adjust for BMI (see Table S7).

Like higher lifetime average alcohol intake, heavy alcohol intake patterns were associated with demographic and lifestyle factors, including being male and being a current smoker (Table S8). In our analysis of alcohol intake patterns, current, consistent heavy drinkers had 91% higher risk of CRC compared with current, consistent light drinkers (HR, 1.91; 95% CI, 1.17–3.12; Table 5). Our results suggest that current drinkers who were moderate drinkers at some point(s) in life and former moderate‐to‐heavy drinkers were not at increased risk of CRC (occasionally moderate vs. always light drinkers: HR, 0.95 [95% CI, 0.81–1.12]; former moderate to heavy vs. always light drinkers: HR, 0.89 [95% CI, 0.69–1.15]). The use of never drinkers as the reference group (see Table S9) did not appreciably alter the findings.

**TABLE 5:** Hazard ratios and 95% confidence intervals for lifetime alcohol intake patterns and incident colorectal cancer.

Drinking status	Drinking frequency	Past range, drinks/week^a^	Current range, drinks/week^b^	Overall no.	Cases, no.	HR (95% CI)^c^
Never	Never drinkers	0	0	10,205	174	0.95 (0.80–1.12)
Former	Former, light	Women, >0 to <7 Men, >0 to <14	0	9469	196	1.09 (0.93–1.28)
	Former, moderate to heavy	At least at one point in time: Women, ≥7 Men, ≥14	0	3740	65	0.89 (0.69–1.15)
Current	Always light, below dietary guidelines^d^	Women, >0 to <7 Men, >0 to <14	Women, >0 to <7 Men, >0 to <14	43,414	812	1.0 (Ref)
	Occasionally moderate	At least at one point in time: Women, ≥7 to ≤14 Men, ≥14 to ≤21		9815	178	0.95 (0.81–1.12)
	Occasionally heavy	At least at one point in time: Women, >14 Men, >21		10,901	233	1.07 (0.92–1.25)
	Always moderate	Women, ≥7 to ≤14 Men, ≥14 to ≤21	Women, ≥7 to ≤14 Men, ≥14 to ≤21	10,901	4	2.08 (0.78–5.57)
	Always heavy	Women, >14 Men, >21	Women, >14 Men, >21	446	17	1.91 (1.17–3.12)

Abbreviations: CI, confidence interval; HR, hazard ratio; Ref, reference group.

^a^ Participants were asked about their alcohol consumption frequency during four predefined age ranges (18–24, 25–39, 40–54, and ≥55 years).

^b^ Participants were asked about their alcohol consumption in the year prior to Dietary History Questionnaire completion. Seventy participants had missing data for current alcohol intake. Therefore, alcohol intake reported for the age range ≥55 years was considered current drinking.

^c^ Adjusted for sex (male or female), age (years), trial arm (intervention or control), race (Asian, Hispanic, Non‐Hispanic Black, Non‐Hispanic White, or other/unknown), college graduate (yes/no), body mass index category (<18.5, 18.5 to <25, 25 to <30, ≥30 kg/m^2^), smoking status (never smoker; cigar/pipe smoker only; former smoker, stopped >20 years ago; former smoker, stopped <20 years ago; current smoker, 0–44 pack‐years; current smoker, ≥44 pack‐years), regular nonsteroidal anti‐inflammatory drug use (yes/no), history of diabetes (yes/no), family history of colon cancer (yes/no), total daily energy (kcal/day), dietary calcium intake (mg/1000 kcal/day), red meat intake (g/1000 kcal/day), dietary fiber intake (g/1000 kcal/day), and dietary folate intake (μg/1000 kcal/day). Global *p* value for lifetime alcohol intake pattern variable, *p* = .12.

^d^ US dietary guidelines for alcohol intake are one drink per day or less for women and two drinks per day or less for men.

## DISCUSSION

In this prospective study, compared with low average lifetime alcohol intake (less than one drink per week), moderate average lifetime alcohol intake (from seven to less than14 drinks per week) was associated with lower CRC risk, specifically distal colon cancer, whereas heavy average lifetime alcohol intake (more than 14 drinks per week) was associated with higher CRC risk, especially rectal cancer. Consistent heavy drinking throughout adulthood was positively associated with CRC risk, whereas occasional moderate or heavy drinking, compared with consistent light drinking below dietary recommendations, was not associated with higher CRC risk. Conclusions were not appreciably changed when never drinkers were used as the reference group. The adenoma results support the CRC findings, highlighting that drinking cessation may lower nonadvanced adenoma risk, whereas heavy drinking may increase adenoma risk. Still, adenoma case numbers were limited in our study; OR estimates were generally not statistically significant; and OR estimates based on fewer than 50 patients should be considered exploratory because of the risk of overfitting and limited precision. The totality of our prospective analyses, including similar results when excluding the first 2 years of follow‐up, suggests that reverse causation is an unlikely explanation for our findings.

Our results are consistent with previous literature. Average lifetime alcohol intake has previously been associated with a modest increase in CRC risk.^8^, ^9^, ^10^ In line with our finding of an inverse association with moderate drinking, a pooled analysis of eight cohort studies (*n* = 489,979, including 4687 patients with CRC and adjusting for smoking status, BMI, education, height, physical activity, family history of CRC, use of nonsteroidal anti‐inflammatory drugs, multivitamin use, energy intake, red meat intake, total milk intake, folate intake, and history of use of oral contraceptives and postmenopausal hormone therapy [women only]) also reported HR estimates <1.0, albeit not statistically significant, for low and moderate current (baseline) drinking compared with nondrinkers. Those authors attributed this to the inclusion of past drinkers in their reference group.^33^ This bias is mitigated in our study because we examined lifetime drinking and evaluated associations for former drinkers. In addition, a recent meta‐analysis of 16 epidemiologic studies indicated that baseline intake less than two alcoholic drinks per day, compared with ≤1 g/day, was associated with lower CRC risk.^7^ Research in rodents suggests that moderate alcohol intake may reduce inflammation and lower DNA damage,^34^ but it is also possible that the observed inverse association is because of residual confounding by unmeasured or poorly measured confounders (e.g., socioeconomic status). In our study, the inverse association of moderate alcohol intake was strongest for distal colon cancer and in the screening arm of the trial. Although the heterogeneity between trial arms was not statistically significant, it is important to note that those in the screening arm who screened positive with flexible sigmoidoscopy had polyps removed and were referred for colonoscopy during the trial period, making screening a potential intervention as well. Screening with flexible sigmoidoscopy has previously been found to decrease CRC incidence, specifically distal colon cancer, in this population.^24^

Similar to our study, Ferrari et al. found that heavier average lifetime alcohol drinking (i.e., ≥30 g/day), compared with light drinking (0.1–4.9 g/day), was associated with higher risk of rectal and distal colon cancers but not proximal colon cancer in a prospective cohort study of about 480,000 European men and women^8^; whereas Thygesen et al. found that a cumulative average alcohol intake of >30 g/day, compared with nondrinkers, was associated with a higher risk for distal colon cancer but not for rectal or proximal colon cancers in a prospective cohort of 47,000 US men.^9^ Differences in assignment of reference group (light drinkers vs. nondrinkers), case numbers by anatomic location (i.e., Thygesen et al. had only 175 rectal cases), and adjustment for prior CRC screening (Thygesen et al. did, whereas Ferrari et al. did not) could account for varying results. However, as in our current study, the site‐specific HR estimates were not statistically different from each other, although such analyses lacked statistical power.

Three prior studies investigated changes in alcohol intake and CRC risk^11^, ^12^, ^13^; however, to the best of our knowledge, none have evaluated alcohol intake patterns throughout adulthood with incident colorectal adenoma. Aligning with our CRC results, Bassett et al. observed that, among men, consistent heavy drinkers, compared with never drinkers, had higher CRC risk^12^; and Mayen et al. reported that men who were consistent moderate‐to‐heavy drinkers, compared with stable low drinkers, throughout adulthood had higher CRC risk.^11^ Mayen et al. also observed that heavy drinkers who decreased alcohol drinking had lower CRC risk, which was similar in direction to the HR estimate for former moderate‐to‐heavy drinkers in our study. In contrast, Yoo et al. reported that former alcohol drinkers (quitters; compared with sustainers), had higher CRC risk.^13^ Those authors attributed this to a sick quitter bias and demonstrated in a sensitivity analysis that former drinkers who remained nondrinking for the next 2 years had similar or lower cancer risk compared with those who sustained drinking levels.^13^

Several potential mechanisms have been proposed to explain the association between alcohol drinking and colorectal neoplasia risk. First, alcohol metabolism produces acetaldehyde, an established carcinogen,^4^, ^35^ which causes DNA mutation and oxidative stress in the colon and rectum.^36^, ^37^, ^38^ Acetaldehyde also hinders folate absorption and disrupts one‐carbon metabolism, potentially leading to abnormalities in DNA methylation, which are common precursor events for colorectal neoplasms.^39^ Second, alcohol drinking may adversely affect the gut microbiome,^36^ resulting in altered host cell proliferation and death^40^, ^41^, ^42^ and immune system function.^43^, ^44^ As mentioned above, there is also some evidence that moderate alcohol intake may reduce inflammation and lower DNA damage.^34^

Our study has notable limitations and strengths. First, our statistical power to detect associations was limited by a relatively small number of cases, particularly in stratified analyses. Second, generalizability may be limited because PLCO participants were predominantly non‐Hispanic White and tended to be healthier and more highly educated than those of a similar age in the US general population.^45^ Future studies could consider pooling data across cohorts with data on lifetime alcohol drinking to increase statistical power and generalizability. Third, multiple testing may have increased the probability of chance findings in our study. However, we did not perform a *p* value correction because our analyses were not independent (e.g., our outcomes are related because adenoma is a precursor of CRC, and each anatomic location is nested within CRC). Fourth, we expect some misclassification of self‐reported alcohol intake (e.g., underreporting because of social desirability bias); however, because of the prospective design of our study, alcohol was reported before the development of disease, so we expect that any bias would be nondifferential with respect to adenoma or CRC incidence. Finally, this is an observational study, and, although we adjusted for potential confounders using detailed questionnaire data, residual confounding by unmeasured or poorly measured factors is possible. Despite these limitations, our study is a key addition to the literature and is novel in its prospective evaluation of the association between lifetime alcohol drinking and the risk of colorectal adenoma and CRC. Alcohol drinking can vary by age, and cumulative exposure over the life course—and not only drinking status in older age—may be an important risk factor for cancer. In addition, our study assessed lifetime alcohol intake before the incidence of colorectal adenoma or CRC, mitigating the risk of selection or reporting bias. Because our study was conducted in a cancer screening trial, we were able to look at both incident adenoma and CRC risk within the same study, allowing us to assess the relation between alcohol and multiple outcomes in the adenoma–carcinoma sequence. Furthermore, by conducting our study within a cancer screening trial, we were able to control for screening through analyses restricted to participants who were randomized to the screening arm (in which there was equal opportunity to be screened).

## CONCLUSION

In conclusion, we observed that current drinkers who had the highest, compared with the lowest, average lifetime intake and consistent heavy drinkers, compared with consistent light drinkers (below dietary guidelines), had higher risks of CRC, particularly for rectal cancer. Current drinkers who had a moderate average lifetime intake, compared with those who had the lowest average lifetime intake, had lower CRC risk, particularly for distal colon cancer. The inverse association with CRC was strongest in the screening arm of the trial, suggesting that screening has the potential to modify the association between alcohol drinking and CRC risk. We also observed that former drinkers had lower nonadvanced adenoma risk. Additional research is needed to understand whether adenoma removal by polypectomy not only lowers CRC risk but also spurs healthy lifestyle changes because our study results suggest that alcohol cessation lowers CRC risk. Future research, including consortium efforts and new cohort studies with data on lifetime alcohol intake, is needed to strengthen evidence on the role of alcohol drinking reduction and cessation in the development of colorectal adenoma and CRC.

## AUTHOR CONTRIBUTIONS

**Caitlin P. O’Connell:** Conceptualization, data curation, methodology, software, formal analysis, writing–original draft, and writing–review and editing. **Sonja I. Berndt:** Conceptualization, methodology, and writing–review and editing. **Kenechukwu Chudy‐Onwugaje:** Formal analysis and writing–review and editing. **Andrew Kunzmann:** Conceptualization and writing–review and editing. **Wen‐Yi Huang:** Conceptualization, methodology, data curation, investigation, writing–review and editing; resources, project administration, and funding acquisition. **Kathryn Hughes Barry:** Conceptualization, writing–original draft, writing–review and editing, supervision, funding acquisition, and project administration. **Erikka Loftfield:** Conceptualization, methodology, formal analysis, software, writing–original draft, writing–review and editing, supervision, and funding acquisition.

## CONFLICT OF INTEREST STATEMENT

The authors disclosed no conflicts of interest.

## Supporting information

Supplementary Material

## Data Availability

The data that support the findings of this study are available on request from the corresponding author. The data are not publicly available because of privacy or ethical restrictions. Data from the Prostate, Lung, Colorectal, and Ovarian (PLCO) Cancer Screening Trial can be shared through a proposal submission and approval process and after an appropriate Data Transfer Agreement is in place. See instructions on access to PLCO data and specimens at https://cdas.cancer.gov/learn/plco/instructions/. Cancer data collected through certain state cancer registries, which comprise approximately 10% of available cancer data overall as of February 2023, have not permitted individual data sharing outside of the National Cancer Institute.
