# Supplementary material for: Association of alcohol intake over the lifetime with colorectal adenoma and colorectal cancer risk in the Prostate, Lung, Colorectal, and Ovarian Cancer Screening Trial
Source: Cancer. 2026 Jan 26;132(3):e70201. doi: 10.1002/cncr.70201 (PMC12833583; doi:10.1002/cncr.70201)
Supplement: Supplementary file 1 — Supplementary Material [file CNCR-132-e70201-s001.docx]

**Supporting Methods**

Average lifetime alcohol consumption was calculated from the following equation, letting Y equal the number of years in each age range, R equal reported daily alcohol consumption in that range, and subscript C indicating current drinking data (i.e., prior 12 months to DHQ):

$$Alcohol_{lifetime}=\frac{Y_{18}R_{18}+Y_{25}R_{25}+Y_{40}R_{40}+Y_{55}R_{55}+Y_{C}R_{C}}{(Y_{18}+Y_{25}+Y_{40}+Y_{55}+Y_{C})}$$

The numerator has units of daily MPED (My Pyramid Equivalents Database) * years, and the denominator is in years. Therefore, average lifetime alcohol drinking represents the average number of alcoholic drinks per day from age 18 to age at DHQ completion in MPED defined servings.

Lifetime alcohol intake patterns were classified by the below table:

| **Drinking status** | **Drinking frequency** | **Past range* (drinks/week)** | **Current range**^†^ **(drinks/week)** |
| --- | --- | --- | --- |
| Never | Never drinkers | 0 | 0 |
| Former | Light | Women: > 0 to < 7  Men: > 0 to < 14 | 0 |
|  | Moderate to heavy | At least at one point in time:  Women: ≥ 7 Men: ≥ 14 | 0 |
| Current | Light | Women: > 0 to < 7  Men: > 0 to < 14 | Women: > 0 to < 7  Men: > 0 to < 14 |
|  | Occasionally moderate | At least at one point in time:  Women: ≥ 7 to ≤ 14  Men: ≥ 14 to ≤ 21 | |
|  | Occasionally heavy | At least at one point in time:  Women: > 14  Men: > 21 | |
|  | Always moderate | Women: ≥ 7 to ≤ 14  Men: ≥ 14 to ≤ 21 | Women: ≥ 7 to ≤ 14  Men: ≥ 14 to ≤ 21 |
|  | Always heavy | Women: > 14  Men: > 21 | Women: > 14  Men: > 21 |

*Participants were asked about their alcohol consumption frequency during four pre-defined age ranges (18-24, 25-39, 40-54, and ≥55 years).

^†^Participants were asked about their alcohol consumption in the year prior to Dietary History Questionnaire completion. In the colorectal cancer analysis, 70 participants had missing data for current alcohol intake. Therefore, alcohol intake reported for the age range ≥55 years was used as current drinking.

**Table S1. Baseline characteristics by lifetime alcohol intake in the PLCO incident adenoma analysis cohort (N=12,327)**

| **Characteristic, n (%)** | **Never drinker** | **Former drinker** | **Current drinker** | | | |
| --- | --- | --- | --- | --- | --- | --- |
| **Lifetime average drinking categories** | **--** | **--** | **<1 drink/week** | **1 to <7 drinks/week** | **7 to <14 drinks/week** | **≥14 drinks/week** |
| **Participants, N** | 1512 | 1774 | 2272 | 4736 | 1195 | 838 |
| **Lifetime median alcohol intake,** drinks/week (IQR) | 0.0 | 0.9 (0.2-6.1) | 0.5 (0.3-0.7) | 2.92 (1.81-4.51) | 9.31 (7.99-11.4) | 19.6 (16.0-26.4) |
| **Age at DHQ**, Median (IQR) | 64.0 (61.0-69.0) | 63.0 (60.0-67.0) | 63.0 (60.0-67.0) | 63.0 (60.0-67.0) | 62.0 (60.0-67.0) | 63.0 (60.0-67.0) |
| **Sex** |  |  |  |  |  |  |
| Male | 467 (30.9%) | 975 (55.0%) | 624 (27.5%) | 2536 (53.5%) | 953 (79.7%) | 764 (91.2%) |
| Female | 1045 (69.1%) | 799 (45.0%) | 1648 (72.5%) | 2200 (46.5%) | 242 (20.3%) | 74 (8.8%) |
| **Race/ethnicity** |  |  |  |  |  |  |
| Asian | 128 (8.5%) | 99 (5.6%) | 158 (7.0%) | 144 (3.0%) | 39 (3.3%) | 41 (4.9%) |
| Hispanic | 14 (0.9%) | 37 (2.1%) | 38 (1.7%) | 85 (1.8%) | 21 (1.8%) | 12 (1.4%) |
| Non-Hispanic Black | 62 (4.1%) | 77 (4.3%) | 69 (3.0%) | 85 (1.8%) | 16 (1.3%) | 24 (2.9%) |
| Non-Hispanic White | 1290 (85.3%) | 1537 (86.6%) | 1992 (87.7%) | 4387 (92.6%) | 1106 (92.6%) | 747 (89.1%) |
| Other/unknown | 18 (1.2%) | 24 (1.4%) | 15 (0.7%) | 35 (0.7%) | 13 (12%) | 14 (1.6%) |
| **College graduate or higher** | 515 (34.1%) | 545 (30.7%) | 912 (40.1%) | 1993 (42.1%) | 508 (42.5%) | 274 (32.7%) |
| **BMI category** |  |  |  |  |  |  |
| <18.5 kg/m2 | 12 (0.8%) | 6 (0.3%) | 12 (0.5%) | 22 (0.5%) | 1 (0.1%) | 2 (0.2%) |
| 18.5 to <25 kg/m2 | 534 (35.3%) | 471 (26.6%) | 822 (36.2%) | 1569 (33.1%) | 317 (26.5%) | 204 (24.3%) |
| 25 to 30 kg/m2 | 582 (38.5%) | 769 (43.3%) | 880 (38.7%) | 2084 (44.0%) | 593 (49.6%) | 414 (49.4%) |
| ≥30 kg/m2 | 375 (24.8%) | 503 (28.4%) | 542 (23.9%) | 1029 (21.7%) | 274 (22.9%) | 209 (24.9%) |
| **Smoking status** |  |  |  |  |  |  |
| Never smoker | 1364 (90.2%) | 790 (44.5%) | 1596 (70.2%) | 2299 (48.5%) | 409 (34.2%) | 213 (25.4%) |
| Former smoker | 127 (8.4%) | 860 (48.5%) | 591 (26.0%) | 2188 (46.2%) | 688 (57.6%) | 521 (62.2%) |
| Current smoker | 21 (1.4%) | 124 (7.0%) | 85 (3.7%) | 249 (5.3%) | 98 (8.2%) | 104 (12.4%) |
| **Family history of colon cancer** | 139 (9.2%) | 142 (8.0%) | 191 (8.4%) | 362 (7.6%) | 88 (7.4%) | 67 (8.0%) |
| **Regular NSAID use** | 653 (43.2%) | 895 (50.5%) | 1003 (44.1%) | 2362 (49.9%) | 670 (56.1%) | 435 (51.9%) |
| **History of diabetes** | 118 (7.8%) | 200 (11.3%) | 98 (4.3%) | 186 (3.9%) | 49 (4.1%) | 44 (5.3%) |
| **HRT use** | 698 (46.2%) | 551 (31.1%) | 1136 (50.0%) | 1585 (33.5%) | 178 (14.9%) | 50 (6.0%) |
| **Median total energy intake**, kcal/day (IQR) | 1520 (1160-1940) | 1600 (1200-2090) | 1470 (1120-1870) | 1670 (1300-2110) | 1940 (1530-2400) | 2190 (1670-2840) |
| **Median dietary calcium intake,** mg/1000 kcal/day (IQR) | 429 (337-564) | 404 (321-525) | 422 (341-542) | 401 (326-509) | 368 (302-448) | 334 (266-426) |
| **Median red meat intake,** g/1000 kcal/day (IQR) | 25.7 (15.5-38.4) | 31.7 (19.3-46.6) | 28.6 (18.0-41.1) | 33.0 (21.5-46.4) | 36.1 (24.9-51.0) | 36.9 (25.8-52.5) |
| **Median dietary fiber intake,** g/1000 kcal/day (IQR) | 11.2 (9.1-13.8) | 10.5 (8.5-13.4) | 11.0 (8.9-13.5) | 10.2 (8.4-12.2) | 9.29 (7.7-11.2) | 8.61 (7.1-10.3) |
| **Median dietary folate intake,** ug/1000 kcal/day (IQR) | 227 (193-270) | 219 (181-262) | 228 (193-269) | 215 (183-254) | 199 (170-236) | 185 (157-218) |

NOTE. Missing values are not shown. Percentages are column percents and do not always add up due to rounding and missing values. Abbreviations: BMI, body mass index; NSAID, nonsteroidal anti-inflammatory drug; HRT, hormone replacement therapy; IQR, interquartile range; DHQ, Dietary History Questionnaire

**Table S2. Hazard ratios (HR) and 95% confidence intervals for average lifetime alcohol intake and incident colorectal adenoma by sex**

|  | Overall | | | | Males | | | | Females | | | |
| --- | --- | --- | --- | --- | --- | --- | --- | --- | --- | --- | --- | --- |
| Alcohol drinking status | Non cases | Cases | OR^*^ | 95% CI | Non cases | Cases | OR^*^ | 95% CI | Non cases | Cases | OR^*^ | 95% CI |
| Never | 1441 | 71 | 0.82 | 0.60-1.10 | 444 | 23 | 0.57 | 0.34-0.94 | 997 | 48 | 1.02 | 0.70-1.50 |
| Former | 1668 | 106 | 0.78 | 0.59-1.02 | 905 | 70 | 0.73 | 0.50-1.08 | 763 | 36 | 0.83 | 0.55-1.25 |
| Current (lifetime average drinks/week) | | | | | | | | | | | | |
| > 0 to <1 | 2139 | 133 | 1.00 | Ref | 570 | 54 | 1.00 | Ref | 1569 | 79 | 1.00 | Ref |
| 1 to <7 | 4424 | 312 | 0.92 | 0.74-1.14 | 2330 | 206 | 0.89 | 0.65-1.23 | 2094 | 106 | 0.89 | 0.65-1.21 |
| 7 to <14 | 1093 | 102 | 1.00 | 0.75-1.34 | 860 | 93 | 1.03 | 0.71-1.48 | 233 | 9 | 0.61 | 0.30-1.26 |
| ≥14 | 750 | 88 | 1.10 | 0.80-1.51 | 683 | 81 | 1.06 | 0.72-1.56 | 67 | 7 | 1.60 | 0.69-3.68 |
| *P* heterogeneity by sex^‡^ |  |  |  | .25 |  |  |  |  |  |  |  |  |

*Adjusted for sex (male or female), age (years), screening year (T3 or T5), race (Asian, Hispanic, Non-Hispanic Black, Non-Hispanic White, or Other/unknown), college graduate (yes/no), BMI category (<18.5, 18.5 to <25, 25 to <30, ≥30+ kg/m^2^), smoking status (never smoker; cigar/pipe smoker only; former smoker, stopped over 20 years ago; former smoker, stopped under 20 years ago; current smoker, 0-44 pack years; current smoker, 44+ pack years), regular NSAID use (yes/no), history of diabetes (yes/no), hormone use (females only, yes/no), family history of colon cancer (yes/no), total daily energy (kcal/day), dietary calcium intake (mg/1000 kcal/day), red meat intake (g/1000 kcal/day), dietary fiber intake (g/1000 kcal/day), and dietary folate intake (ug/1000 kcal/day).

^‡^Estimated by likelihood ratio test comparing models with and without an interaction term between sex and average lifetime alcohol intake.

**Table S3. Odds ratios (OR) and 95% confidence intervals (CI) for lifetime alcohol intake patterns and incident adenoma, with never drinkers as the reference category**

| **Drinking status** | **Drinking frequency** | **Past range* (drinks/week)** | **Current range**^†^ **(drinks/week)** | **Non case** | **Case** | **OR**^‡^ **(95% CI)** |
| --- | --- | --- | --- | --- | --- | --- |
| Never | Never | 0 | 0 | 1441 | 71 | 1.00 (ref) |
| Former | Former | Any value | 0 | 1669 | 106 | 0.96 (0.69-1.33) |
| Current | Always light (below dietary guidelines^§^) | Women: > 0 to < 7  Men: > 0 to < 14 | Women: > 0 to < 7  Men: > 0 to < 14 | 5896 | 407 | 1.16 (0.88-1.52) |
|  | Occasionally moderate | At least at one point in time:  Women: ≥ 7 to ≤ 14  Men: ≥ 14 to ≤ 21 | | 1214 | 96 | 1.23 (0.88-1.52) |
|  | Occasionally heavy | At least at one point in time:  Women: > 14  Men: > 21 | | 1236 | 125 | 1.31 (0.94-1.83) |
|  | Always moderate or heavy | Women: ≥ 7  Men: ≥ 14 | Women: ≥ 7  Men: ≥ 14 | 59 | 7 | 1.46 (0.62-3.42) |

*Participants were asked about frequency of consumption of alcohol during four pre-defined age ranges (18-24, 25-39, 40-54, and ≥55 years).

^†^Participants were asked about their alcohol consumption in the year prior to Dietary History Questionnaire completion.

^‡^Adjusted for sex (male or female), age (years), screening year (T3 or T5), race (Asian, Hispanic, Non-Hispanic Black, Non-Hispanic White, or Other/unknown), college graduate (yes/no), BMI category (<18.5, 18.5 to <25, 25 to <30, ≥30+ kg/m^2^), smoking status (never smoker; cigar/pipe smoker only; former smoker, stopped over 20 years ago; former smoker, stopped under 20 years ago; current smoker, 0-44 pack years; current smoker, 44+ pack years), regular NSAID use (yes/no), history of diabetes (yes/no), family history of colon cancer (yes/no), total daily energy (kcal/day), dietary calcium intake (mg/1000 kcal/day), red meat intake (g/1000 kcal/day), dietary fiber intake (g/1000 kcal/day), and dietary folate intake (ug/1000 kcal/day). Global *P*-value for lifetime alcohol intake pattern variable =.25.

^§^US dietary guidelines for alcohol intake are 1 drink/day or less for women and 2 drinks/day or less for men.

**Table S4. Hazard ratios (HR) and 95% confidence intervals for average lifetime alcohol intake and incident colorectal cancer by randomization arm of the screening intervention trial**

|  | Overall | | | | Screening arm | | | | Control arm | | | |
| --- | --- | --- | --- | --- | --- | --- | --- | --- | --- | --- | --- | --- |
| Alcohol drinking status | Person-years | Cases | HR* | 95% CI | Person-years | Cases | HR* | 95% CI | Person-years | Cases | HR* | 95% CI |
| Never | 147,929 | 174 | 0.92 | 0.76-1.11 | 75,753 | 78 | 0.88 | 0.66-1.17 | 72,175 | 96 | 0.96 | 0.74-1.24 |
| Former | 181,050 | 261 | 1.00 | 0.84-1.19 | 93,148 | 108 | 0.91 | 0.70-1.18 | 87,902 | 153 | 1.09 | 0.86-1.37 |
| Current (lifetime average drinks/week) | | | | | | | | | | | | |
| > 0 to <1 | 234,828 | 286 | 1.00 | Ref | 118,506 | 131 | 1.00 | Ref | 116,322 | 155 | 1.00 | Ref |
| 1 to <7 | 491,379 | 631 | 0.97 | 0.84-1.13 | 255,918 | 272 | 0.92 | 0.74-1.15 | 235,444 | 359 | 1.02 | 0.83-1.23 |
| 7 to <14 | 130,091 | 149 | **0.79** | **0.64-0.97** | 67,575 | 59 | **0.71** | **0.51-0.98** | 62,549 | 90 | 0.85 | 0.65-1.12 |
| ≥14 | 91,288 | 178 | **1.25** | **1.01-1.53** | 47,512 | 66 | 1.07 | 0.77-1.48 | 43,760 | 112 | **1.39** | **1.06-1.82** |
| *P* heterogeneity by arm^‡^ |  |  |  | .33 |  |  |  |  |  |  |  |  |

*Adjusted for sex (male or female), age (years), race (Asian, Hispanic, Non-Hispanic Black, Non-Hispanic White, or Other/unknown), college graduate (yes/no), BMI category (<18.5, 18.5 to <25, 25 to <30, ≥30+ kg/m^2^), smoking status (never smoker; cigar/pipe smoker only; former smoker, stopped over 20 years ago; former smoker, stopped under 20 years ago; current smoker, 0-44 pack years; current smoker, 44+ pack years), regular NSAID use (yes/no), history of diabetes (yes/no), family history of colon cancer (yes/no), total daily energy (kcal/day), dietary calcium intake (mg/1000 kcal/day), red meat intake (g/1000 kcal/day), dietary fiber intake (g/1000 kcal/day), and dietary folate intake (ug/1000 kcal/day). Overall estimate is also adjusted for trial arm.

^‡^Estimated by likelihood ratio test comparing models with and without an interaction term between study arm and average lifetime alcohol intake variable.

**Table S5. Hazard ratios (HR) and 95% confidence intervals for average lifetime alcohol intake and incident colorectal cancer by sex**

|  | Overall | | | | Males | | | | Females | | | |
| --- | --- | --- | --- | --- | --- | --- | --- | --- | --- | --- | --- | --- |
| Alcohol drinking status | Person-years | Cases | HR* | 95% CI | Person-years | Cases | HR* | 95% CI | Person-years | Cases | HR* | 95% CI |
| Never | 147,929 | 174 | 0.92 | 0.76-1.11 | 44,419 | 55 | 0.88 | 0.62-1.25 | 103,510 | 119 | 0.96 | 0.76-1.20 |
| Former | 181,050 | 261 | 1.00 | 0.84-1.19 | 59,468 | 128 | 0.93 | 0.70-1.24 | 91,582 | 133 | 1.12 | 0.90-1.40 |
| Current (lifetime average drinks/week) | | | | | | | | | | | | |
| > 0 to <1 | 234,828 | 286 | 1.00 | Ref | 54,295 | 76 | 1.00 | Ref | 180,532 | 210 | 1.00 | Ref |
| 1 to <7 | 491,379 | 631 | 0.97 | 0.84-1.13 | 246,240 | 377 | 1.08 | 0.85-1.39 | 245,121 | 254 | 0.87 | 0.72-1.05 |
| 7 to <14 | 130,091 | 149 | **0.79** | **0.64-0.97** | 96,897 | 114 | 0.80 | 0.59-1.07 | 33,228 | 35 | 0.86 | 0.59-1.24 |
| ≥14 | 91,288 | 178 | **1.25** | **1.01-1.53** | 81,404 | 162 | 1.28 | 0.96-1.70 | 9,868 | 16 | 1.30 | 0.77-2.18 |
| *P* heterogeneity by sex^‡^ |  |  |  | .10 |  |  |  |  |  |  |  |  |

*Adjusted for sex (male or female), age (years), race (Asian, Hispanic, Non-Hispanic Black, Non-Hispanic White, or Other/unknown), college graduate (yes/no), BMI category (<18.5, 18.5 to <25, 25 to <30, ≥30+ kg/m^2^), smoking status (never smoker; cigar/pipe smoker only; former smoker, stopped over 20 years ago; former smoker, stopped under 20 years ago; current smoker, 0-44 pack years; current smoker, 44+ pack years), regular NSAID use (yes/no), history of diabetes (yes/no), family history of colon cancer (yes/no), total daily energy (kcal/day), dietary calcium intake (mg/1000 kcal/day), red meat intake (g/1000 kcal/day), dietary fiber intake (g/1000 kcal/day), and dietary folate intake (ug/1000 kcal/day). Overall estimate is also adjusted for trial arm.

^‡^Estimated by likelihood ratio test comparing models with and without an interaction term between sex and average lifetime alcohol intake.

**Table S6. Hazard ratios (HR) and 95% confidence intervals for average lifetime alcohol intake and incident colorectal cancer excluding first two years of follow-up**

|  | Overall | | | | Screening arm | | | Control arm | | |
| --- | --- | --- | --- | --- | --- | --- | --- | --- | --- | --- |
| Alcohol drinking status | Person-years | Cases | HR* | 95% CI | Cases | HR* | 95% CI | Cases | HR* | 95% CI |
| Never | 147,740 | 146 | 0.91 | 0.74-1.13 | 67 | 0.86 | 0.63-1.17 | 79 | 0.96 | 0.72-1.28 |
| Former | 180,590 | 221 | 1.02 | 0.85-1.23 | 93 | 0.92 | 0.69-1.21 | 128 | 1.12 | 0.87-1.44 |
| Current (lifetime average drinks/week) | | | | | | | | | | |
| > 0 to <1 | 234,588 | 242 | 1.00 | Ref | 114 | 1.00 | Ref | 128 | 1.00 | Ref |
| 1 to <7 | 490,837 | 556 | 1.02 | 0.87-1.19 | 247 | 0.97 | 0.77-1.22 | 309 | 1.06 | 0.86-1.31 |
| 7 to <14 | 129,887 | 128 | 0.81 | 0.64-1.01 | 52 | 0.73 | 0.51-1.03 | 76 | 0.87 | 0.65-1.18 |
| ≥14 | 90,995 | 152 | **1.28** | **1.02-1.60** | 57 | 1.09 | 0.77-1.55 | 95 | **1.43** | **1.07-1.93** |
| *P* value, interaction by trial arm^‡^ |  |  | .33 |  |  |  |  |  |  |  |

*Adjusted for sex (male or female), age (years), trial arm (intervention or control), race (Asian, Hispanic, Non-Hispanic Black, Non-Hispanic White, or Other/unknown), college graduate (yes/no), BMI category (<18.5, 18.5 to <25, 25 to <30, ≥30+ kg/m^2^), smoking status (never smoker; cigar/pipe smoker only; former smoker, stopped over 20 years ago; former smoker, stopped under 20 years ago; current smoker, 0-44 pack years; current smoker, 44+ pack years), regular NSAID use (yes/no), history of diabetes (yes/no), hormone use (females only, yes/no), family history of colon cancer (yes/no), total daily energy (kcal/day), dietary calcium intake (mg/1000 kcal/day), red meat intake (g/1000 kcal/day), dietary fiber intake (g/1000 kcal/day), and dietary folate intake (ug/1000 kcal/day).

^‡^Estimated by likelihood ratio test comparing models with and without an interaction term between trial arm and alcohol exposure variable.

**Table S7. Hazard ratios (HR) and 95% confidence intervals for average lifetime alcohol intake and incident colorectal cancer without adjustment for body mass index (BMI)**

| Alcohol drinking status | Person-years | Cases | HR* | 95% CI |
| --- | --- | --- | --- | --- |
| Never | 147,740 | 146 | 0.92 | 0.76-1.12 |
| Former | 180,590 | 221 | 1.01 | 0.85-1.20 |
| Current (lifetime average drinks/week) | | | | |
| > 0 to <1 | 234,588 | 242 | 1.00 | Ref |
| 1 to <7 | 490,837 | 556 | 0.96 | 0.83-1.11 |
| 7 to <14 | 129,887 | 128 | **0.78** | **0.63-0.96** |
| ≥14 | 90,995 | 152 | **1.24** | **1.01-1.52** |

*Adjusted for sex (male or female), age (years), trial arm (intervention or control), race (Asian, Hispanic, Non-Hispanic Black, Non-Hispanic White, or Other/unknown), college graduate (yes/no), smoking status (never smoker; cigar/pipe smoker only; former smoker, stopped over 20 years ago; former smoker, stopped under 20 years ago; current smoker, 0-44 pack years; current smoker, 44+ pack years), regular NSAID use (yes/no), history of diabetes (yes/no), hormone use (females only, yes/no), family history of colon cancer (yes/no), total daily energy (kcal/day), dietary calcium intake (mg/1000 kcal/day), red meat intake (g/1000 kcal/day), dietary fiber intake (g/1000 kcal/day), and dietary folate intake (ug/1000 kcal/day).

**Table S8. Baseline characteristics by lifetime alcohol intake patterns in the PLCO incident colorectal cancer analysis cohort (N=88,092)**

|  | **Never drinkers (N=10205)** | **Former - light (N=9469)** | **Former - moderate to heavy (N=3740)** | **Always light (N=43414)** | **Occasionally moderate (N=9815)** | **Occasionally heavy (N=10901)** | **Always moderate (N=102)** | **Always heavy (N=446)** |
| --- | --- | --- | --- | --- | --- | --- | --- | --- |
| **Age at DHQ**, Median (IQR) | 67.0 (62.0- 71.0) | 66.0 (61.0- 70.0) | 65.0 (61.0- 69.0) | 65.0 (60.0- 70.0) | 65.0 (61.0- 70.0) | 65.0 (60.0- 69.0) | 64.5 (59.3- 68.8) | 64.0 (60.0- 68.0) |
| **Sex** |  |  |  |  |  |  |  |  |
| Male | 3191 (31.3%) | 3989 (42.1%) | 2791 (74.6%) | 20948 (48.3%) | 4984 (50.8%) | 7406 (67.9%) | 65 (63.7%) | 394 (88.3%) |
| Female | 7014 (68.7%) | 5480 (57.9%) | 949 (25.4%) | 22466 (51.7%) | 4831 (49.2%) | 3495 (32.1%) | 37 (36.3%) | 52 (11.7%) |
| **Race/ethnicity** |  |  |  |  |  |  |  |  |
| Asian | 805 (7.9%) | 494 (5.2%) | 87 (2.3%) | 1362 (3.1%) | 212 (2.2%) | 289 (2.7%) | 3 (2.9%) | 16 (3.6%) |
| Hispanic | 95 (0.9%) | 133 (1.4%) | 65 (1.7%) | 627 (1.4%) | 133 (1.4%) | 156 (1.4%) | 0 (0%) | 6 (1.3%) |
| Non-Hispanic Black | 543 (5.3%) | 569 (6.0%) | 191 (5.1%) | 1221 (2.8%) | 175 (1.8%) | 307 (2.8%) | 1 (1.0%) | 16 (3.6%) |
| Non-Hispanic White | 8669 (84.9%) | 8184 (86.4%) | 3355 (89.7%) | 39954 (92.0%) | 9239 (94.1%) | 10053 (92.2%) | 95 (93.1%) | 402 (90.1%) |
| Other/unknown | 93 (0.9%) | 89 (0.9%) | 42 (1.1%) | 250 (0.6%) | 56 (0.6%) | 96 (0.9%) | 3 (2.9%) | 6 (1.3%) |
| **College graduate or higher** | 3497 (34.3%) | 2798 (29.5%) | 1123 (30.0%) | 17984 (41.4%) | 4204 (42.8%) | 4122 (37.8%) | 36 (35.3%) | 103 (23.1%) |
| **BMI category** |  |  |  |  |  |  |  |  |
| <18.5 kg/m2 | 101 (1.0%) | 73 (0.8%) | 27 (0.7%) | 231 (0.5%) | 79 (0.8%) | 72 (0.7%) | 1 (1.0%) | 1 (0.2%) |
| 18.5 to <25 kg/m2 | 3548 (34.8%) | 2775 (29.3%) | 1004 (26.8%) | 14702 (33.9%) | 3815 (38.9%) | 3541 (32.5%) | 39 (38.2%) | 123 (27.6%) |
| 25 to 30 kg/m2 | 3928 (38.5%) | 3791 (40.0%) | 1671 (44.7%) | 18535 (42.7%) | 4101 (41.8%) | 4787 (43.9%) | 51 (50.0%) | 210 (47.1%) |
| ≥30 kg/m2 | 2512 (24.6%) | 2733 (28.9%) | 978 (26.1%) | 9415 (21.7%) | 1718 (17.5%) | 2359 (21.6%) | 11 (10.8%) | 110 (24.7%) |
| **Smoking status** |  |  |  |  |  |  |  |  |
| Never smoker | 8811 (86.3%) | 4601 (48.6%) | 605 (16.2%) | 21731 (50.1%) | 3191 (32.5%) | 2423 (22.2%) | 22 (21.6%) | 68 (15.2%) |
| Former smoker | 1119 (11.0%) | 4028 (42.5%) | 2429 (64.9%) | 18227 (42.0%) | 5622 (57.3%) | 6653 (61.0%) | 63 (61.8%) | 277 (62.1%) |
| Current smoker | 274 (2.7%) | 840 (8.9%) | 704 (18.8%) | 3452 (8.0%) | 1002 (10.2%) | 1823 (16.7%) | 17 (16.7%) | 101 (22.6%) |
| **Family history of colon cancer** | 1040 (10.2%) | 1038 (11.0%) | 324 (8.7%) | 4358 (10.0%) | 973 (9.9%) | 1003 (9.2%) | 12 (11.8%) | 47 (10.5%) |
| **Regular NSAID use** | 4477 (43.9%) | 4561 (48.2%) | 2061 (55.1%) | 21486 (49.5%) | 5197 (52.9%) | 5650 (51.8%) | 52 (51.0%) | 218 (48.9%) |
| **History of diabetes** | 988 (9.7%) | 1240 (13.1%) | 447 (12.0%) | 2216 (5.1%) | 367 (3.7%) | 650 (6.0%) | 4 (3.9%) | 26 (5.8%) |
| **Hormone use** | 4496 (44.1%) | 3500 (37.0%) | 649 (17.4%) | 15548 (35.8%) | 3465 (35.3%) | 2467 (22.6%) | 28 (27.5%) | 35 (7.8%) |
| **Median total energy intake**, kcal/day (IQR) | 1470 (1120- 1920) | 1520 (1150- 1970) | 1800 (1350- 2400) | 1560 (1200- 2020) | 1710 (1340- 2180) | 1990 (1530- 2570) | 2000 (1580- 2270) | 2650 (2110- 3590) |
| **Median dietary calcium intake,** mg/1000 kcal/day (IQR) | 430 (337- 567) | 416 (330- 544) | 401 (321- 518) | 411 (330- 528) | 390 (316- 493) | 346 (277- 442) | 388 (314- 453) | 283 (223- 357) |
| **Median red meat intake,** g/1000 kcal/day (IQR) | 25.4 (15.2- 38.4) | 28.5 (17.4- 43.0) | 34.7 (21.8- 51.0) | 31.0 (19.9- 44.6) | 31.2 (20.1- 45.4) | 33.3 (21.8- 47.7) | 31.6 (18.7- 44.8) | 32.4 (20.7- 44.7) |
| **Median dietary fiber intake,** g/1000 kcal/day (IQR) | 11.1 (9.0- 13.8) | 10.8 (8.7- 13.5) | 9.73 (7.6- 12.2) | 10.4 (8.5- 12.7) | 9.74 (8.1- 11.9) | 8.73 (7.1- 10.7) | 9.47 (8.2- 11.6) | 8.00 (6.4- 9.4) |
| **Median dietary folate intake,** ug/1000 kcal/day (IQR) | 228 (192- 273) | 225 (187- 270) | 205 (169- 250) | 220 (186- 261) | 209 (176- 249) | 189 (158- 227) | 207 (172- 241) | 165 (140- 196) |
| **Trial arm** |  |  |  |  |  |  |  |  |
| Screening | 5351 (52.4%) | 4930 (52.1%) | 1988 (53.2%) | 22767 (52.4%) | 5127 (52.2%) | 5673 (52.0%) | 45 (44.1%) | 241 (54.0%) |
| Control | 4854 (47.6%) | 4539 (47.9%) | 1752 (46.8%) | 20647 (47.6%) | 4688 (47.8%) | 5228 (48.0%) | 57 (55.9%) | 205 (46.0%) |

NOTE. Missing values are not shown. Percentages are column percents and do not always add up due to rounding and missing values.

Abbreviations: BMI, body mass index; NSAID, nonsteroidal anti-inflammatory drug; IQR, interquartile range; DHQ, Dietary History Questionnaire

**Table S9. Hazard ratios (HR) and 95% confidence intervals for lifetime alcohol intake patterns and incident colorectal cancer, with never drinkers as the reference category**

| **Drinking status** | **Drinking frequency** | **Past range* (drinks/week)** | **Current range**^†^ **(drinks/week)** | **Overall** | **Case** | **HR**^‡^ **(95% CI)** |
| --- | --- | --- | --- | --- | --- | --- |
| Never | Never drinkers | 0 | 0 | 10205 | 174 | 1.0 (ref) |
| Former | Former - light | Women: > 0 to < 7  Men: > 0 to < 14 | 0 | 9469 | 196 | 1.15 (0.93-1.42) |
|  | Former - moderate to heavy | At least at one point in time:  Women: ≥7 Men: ≥14 | 0 | 3740 | 65 | 0.94 (0.70-1.26) |
| Current | Always light (below dietary guidelines^§^) | Women: > 0 to < 7  Men: > 0 to < 14 | Women: > 0 to < 7  Men: > 0 to < 14 | 43414 | 812 | 1.06 (0.89-1.26) |
|  | Occasionally moderate | At least at one point in time:  Women: ≥ 7 to ≤ 14  Men: ≥ 14 to ≤ 21 | | 9815 | 178 | 1.01 (0.81-1.26) |
|  | Occasionally heavy | At least at one point in time:  Women: > 14  Men: > 21 | | 10901 | 233 | 1.13 (0.91-1.41) |
|  | Always moderate | Women: ≥ 7 to ≤ 14  Men: ≥ 14 to ≤ 21 | Women: ≥ 7 to ≤ 14  Men: ≥ 14 to ≤ 21 | 10901 | 4 | 2.20 (0.81-5.95) |
|  | Always heavy | Women: > 14  Men: > 21 | Women: > 14  Men: > 21 | 446 | 17 | **2.02 (1.21-3.38)** |

*Participants were asked about their alcohol consumption frequency during four pre-defined age ranges (18-24, 25-39, 40-54, and ≥55 years).

^†^Participants were asked about their alcohol consumption in the year prior to Dietary History Questionnaire completion. 70 participants had missing data for current alcohol intake. Therefore, alcohol intake reported for the age range ≥55 years was used as current drinking.

^‡^Adjusted for sex (male or female), age (years), trial arm (intervention or control), race (Asian, Hispanic, Non-Hispanic Black, Non-Hispanic White, or Other/unknown), college graduate (yes/no), BMI category (<18.5, 18.5 to <25, 25 to <30, ≥30+ kg/m^2^), smoking status (never smoker; cigar/pipe smoker only; former smoker, stopped over 20 years ago; former smoker, stopped under 20 years ago; current smoker, 0-44 pack years; current smoker, 44+ pack years), regular NSAID use (yes/no), history of diabetes (yes/no), family history of colon cancer (yes/no), total daily energy (kcal/day), dietary calcium intake (mg/1000 kcal/day), red meat intake (g/1000 kcal/day), dietary fiber intake (g/1000 kcal/day), and dietary folate intake (ug/1000 kcal/day). Global *P*-value for lifetime alcohol intake pattern variable =.12.

^§^US dietary guidelines for alcohol intake are 1 drink/day or less for women and 2 drinks/day or less for men.
